# Cystatin M/E (Cystatin 6): A Janus-Faced Cysteine Protease Inhibitor with Both Tumor-Suppressing and Tumor-Promoting Functions

**DOI:** 10.3390/cancers13081877

**Published:** 2021-04-14

**Authors:** Gilles Lalmanach, Mariana Kasabova-Arjomand, Fabien Lecaille, Ahlame Saidi

**Affiliations:** 1 Faculté de Médecine, Université de Tours, 37000 Tours, France; fabien.lecaille@univ-tours.fr (F.L.); ahlame.saidi@univ-tours.fr (A.S.); 2 Institut National de la Santé et de Recherche Médicale (INSERM), UMR 1100, Research Centre for Respiratory Diseases (CEPR), Team “Proteolytic Mechanisms in Inflammation”, 37000 Tours, France; 3 Covance CAPS Ltd., 1404 Sofia, Bulgaria; mariana.kasabova@gmail.com

**Keywords:** biomarker, breast cancer, cathepsin, cystatin, cysteine protease, legumain, protease inhibitor, tumorigenesis

## Abstract

**Simple Summary:**

Cystatin M/E is a low molecular mass protein and a potent inhibitor of proteolytic enzymes (cathepsins B, L, V, and legumain). Cystatin M/E participates in epidermal homeostasis and its deregulation is associated with several skin diseases. Cystatin M/E was initially identified as a tumor suppressor in breast cancer. Its gene is epigenetically downregulated and its methylation status holds a prognostic significance. Moreover, it may also serve as a biomarker for clinical diagnosis. This tumor-suppressing role was documented in cutaneous carcinoma, melanoma, lung, cervical, brain, prostate, gastric and renal cancers. Conversely, increased levels of cystatin M/E in triple-negative breast cancer tissues correlate with a higher risk of metastasis and a lower disease-free survival rate. Beside its orthodox tumor-suppressing role, cystatin M/E may operate as a tumor-promoting effector as reported for thyroid, oral and pancreatic cancer and hepatocellular carcinoma. Given its seemingly contradictory results, in-depth analysis of the regulatory mechanisms of the expression and activity of cystatin M/E during tumorigenesis have to be expanded. Likewise, the possible involvement of cystatin M/E in signaling pathways, beside its protease inhibitor function, remains to be scrutinized.

**Abstract:**

Alongside its contribution in maintaining skin homeostasis and its probable involvement in fetal and placental development, cystatin M/E (also known as cystatin 6) was first described as a tumor suppressor of breast cancer. This review aims to provide an update on cystatin M/E with particular attention paid to its role during tumorigenesis. Cystatin M/E, which is related to type 2 cystatins, displays the unique property of being a dual tight-binding inhibitor of both legumain (also known as asparagine endopeptidase) and cysteine cathepsins L, V and B, while its expression level is epigenetically regulated via the methylation of the *CST6* promoter region. The tumor-suppressing role of cystatin M/E was further reported in melanoma, cervical, brain, prostate, gastric and renal cancers, and cystatin M/E was proposed as a biomarker of prognostic significance. Contrariwise, cystatin M/E could have an antagonistic function, acting as a tumor promoter (e.g., oral, pancreatic cancer, thyroid and hepatocellular carcinoma). Taking into account these apparently divergent functions, there is an urgent need to decipher the molecular and cellular regulatory mechanisms of the expression and activity of cystatin M/E associated with the safeguarding homeostasis of the proteolytic balance as well as its imbalance in cancer.

## 1. Introduction: General Characteristics of Cystatins

Cystatins are reversible and competitive inhibitors of cysteine proteases operating both extracellularly and intracellularly (for review: [1,2]). Based on structural and functional relationships, cystatins (12 members in humans) belong to family I25 (clan IH) [3]. They are classified into three main subfamilies: stefins (type 1, subfamily I25A), cystatins (type 2, subfamily I25B) and kininogens (type 3, subfamily I25C). Most cystatins are tight-binding and reversible inhibitors of human cysteine cathepsins B, C, F, H, K, L, O, S, V, W and X (clan CA, family C1) that are related to papain from Carica papaya [4,5,6]. Cystatins interact with their target enzymes via three conserved regions in the inhibitor: an N-terminal substrate-like segment, a central QXVXG consensus pentapeptide that stabilizes the complex via an area of extended contact with the protease and a C-terminal region containing a “PW” pair (see for review: [7]). These three regions are spatially continuous and form a wedge-shaped edge, which is highly complementary to the active site of the enzyme [8,9]. Additionally, cystatins C, F and M/E are potent inhibitors of legumain (clan CA, family C13) [10,11]. According to their diverse and wide-ranging distribution, type 1 cystatins A and B (also known as stefins A and B) are primarily cytosolic molecules, but they are likely present in various body fluids [12]. Type 1 cystatins are non-glycosylated single-chain proteins (circa 100 amino acids, 11 kDa) and neither contain disulfide bonds. Human kininogens (i.e., HK, high molecular weight kininogen, approx. 90–120 kDa and LK, low molecular weight kininogen, approx. 50–70 kDa) were initially identified as precursors of pro-inflammatory kinins and players of the coagulation cascade [13], as well as effective inhibitors of cysteine cathepsins (Ki: pM–nM range) [14]. Both kininogens are multidomain glycosylated proteins that encompass an N-terminal heavy chain and a C-terminal light chain interconnected by a disulfide bridge. Heavy chains are identical and encompass three tandemly repeated cystatin-like domains linked to a nonapeptide corresponding to bradykinin, a proinflammatory and vasodilator hormone (for review: [15]). The light chain of LK consists of a single domain while it embraces two distinct domains in HK. Kininogens are synthesized primarily by hepatocytes and are found in various biological fluids (blood plasma, urine, sperm, synovial and amniotic fluids), as well in organs (liver, lung and spleen) and in white blood cells. Like stefins, type 2 cystatins (cystatins C, D, M/E, F, G, S, SN and SA) are single-chain polypeptides of approximately 120 amino acids (circa 13 kDa). They display two conserved disulfide bridges, except cystatin F that exhibits an additional third disulfide bond. Primary sequences of human cystatins C, D, S, SA and SN exhibit more than 50% identities, while cystatins M/E, F and G show lower sequence identities (<35%). Moreover, cystatins M/E and F are glycosylated while cystatins S, SA and SN may be phosphorylated [2]. They are synthesized with 20–26 residue long signal peptides, which support a primarily extracellular localization [1], despite the potential cellular uptake of cystatins by human epithelial cells [16,17]. Accordingly, secreted type 2 cystatins are mainly uncovered in nearly all biological fluids [18]. Cystatin C, the best characterized member of the subfamily I25B, has a widespread distribution and is found in all body fluids, especially in seminal plasma and in cerebrospinal fluid [12]. Low levels of cystatin M/E are detected in mucosal secretions and biological fluids, including human tears and breast milk, and are almost absent or below detection limits in saliva, urine and nasal secretions of healthy volunteers [19]. Conversely, elevated amounts of immunoreactive cystatin M/E are found in seminal plasma (approx. 500 ng/mL). Cystatins S, SA and SN, which are specialized glandular cystatins with restricted expression patterns, are primarily detected in saliva, tears, urine and seminal plasma. Cystatin D is secreted by the parotid gland and is also present in saliva and tears [18]. Cystatin F (also known as leukocystatin) is mainly found in spleen and in immune cells [20]; its concentration in body fluids is generally low, except blood serum and pleural fluid from patients with inflammatory lung disorders [21,22]. Unlike other type 2 cystatins, significant amounts of cystatin F are detected intracellularly, supporting its presumed role in the regulation of proteolytic pathways involved in antigen presentation. Unrelated to their function of protease inhibitors, cystatins may act as potent immunomodulators [23]. Indeed, various cystatins induce the synthesis of TNF-α and interleukin 10, which in turn upregulate the production of NO by IFN-γ-activated macrophages [24,25]. Type 1 cystatins as well as cystatin C may also participate in the regulation of apoptosis [26].

## 2. Gene and Protein Structures of Cystatin M/E

### 2.1. CST6

Almost simultaneously, cystatin M was characterized as the product of a downregulated gene in breast cancer [27], while Abrahamson and colleagues first identified cystatin E as a cDNA corresponding to a rare mRNA species in epithelial cells [28]. The nucleotide sequence encoded human pre-cystatin E and showed the presence of an open reading frame containing a typical consensus sequence for the initiation of translation around the start ATG codon, followed by a poly(A) signal, 78 nucleotides downstream from the stop codon, after which a poly(A) sequence was evident a further 20 nucleotides downstream. Northern blot analysis indicated that the distribution pattern was deeply different from the distribution of cystatin C mRNA and that the cystatin E gene was expressed ubiquitously in the uterus, liver, placenta, pancreas, heart, spleen, small intestine and blood leucocytes, and in a lesser amount in the brain, testis and kidney. Moreover, all cDNA clones originated either from amniotic membrane cells or fetal skin epithelium libraries, which suggests that its gene expression is strongly upregulated in, or specific for, epithelial cells. The authors proposed that cystatin E may play a protective role during fetal development [28]. Alternatively, a partial cDNA of cystatin M was pinpointed to be expressed specifically in the primary tumor cell line but not in metastatic breast tumor cells. The authors identified a full-length cystatin M cDNA containing an open reading frame of 447 nucleotides (24–470), a short 5′-untranslated sequence (1–23) and a 3′-untranslated sequence of 128 nucleotides, with a polyadenylation signal AATAAA (552–557) and a poly(A) tail. Besides its expression by normal mammary cells, consistent levels of cystatin M mRNA were distributed in the placenta, lung, skeletal muscle, kidney and pancreas [27]. These twin papers paved the way for further successful research on cystatin M/E, also known as cystatin 6.

The gene encoding cystatin M/E (*CST6*) is located on the 11q13 region, while the cystatin C gene (*CST3*) is located on 20p11.2 [29]. The complete sequence of the human *CST6* gene was first elucidated by Zeuween et al. [30]. Briefly, *CST6* encompasses three exons within ~1.4 kb of genomic sequence from the translation start site to the translation termination site and a specific genomic organization within the cystatin 2 family, since the TATA box is lacking in the 5′-flanking sequence. Additionally, the two introns of *CST6* (542 bp and 365 bp) are remarkably short compared to introns of cystatins C, D or S genes (respectively, 1.1 to 2.2 kb). The mouse *CST6* ortholog was characterized by the same group and was assigned to the proximal end of chromosome 19. Mouse and human *CST6* retain 69% amino acid identity and 82% amino acid conservation [31].

### 2.2. Protein Structure

Cystatin M/E is synthesized with a signal peptide (1–28) and a main peptide chain (29–149). Mature cystatin M/E is composed of 121 amino acid residues (theoretical pI = 6.78). Its sequence has some unusual characteristics compared to cystatin C, the archetypal member of type 2 cystatins, specifically a five-residue insertion between amino acids 76 and 77, and a deletion of residue 91 (cystatin C numbering). Likewise, cystatin M/E displays two disulfide bridges at positions 73–83 and 97–117, respectively, and an N-glycosylation site at Asn108 [28]. Cystatin M/E is found both in a ~14 kDa non-glycosylated form and a ~17 kDa glycosylated form, and carbohydrates correspond to a complex mannose-linked type [32,33].

In a preliminary study, the three-dimensional structure of cystatin M/E was generated by homology modelling and refined by molecular dynamics simulation. The N-terminal region of cystatin M/E contributes equally to the inhibition of papain together with the conserved L1 and L2 loops [34]. Alternatively, a three-dimensional model of human cystatin M/E was generated on the basis of the crystal structure of human cystatin D, supporting that the overall predicted structure of cystatin M/E narrowly matches that of cystatin D [35]. Moreover, bioinformatics data support that legumain and cysteine cathepsins are inhibited by two distinct non-overlapping binding sites. Finally, the three-dimensional crystal structure of cystatin M/E was solved by Brandstetter and collaborators. Cystatin M/E is characterized by a typical 5-stranded anti-parallel β-sheet that is wrapped around an almost central, perpendicular five-turn α-helix. Two disulfide bridges additionally stabilize the structure by clamping strands β4 and β5 and the cystatin M/E-specific appending structure that is inserted between strands β3 and β4 [36].

## 3. Inhibitory Function 

### 3.1. Cysteine Cathepsins 

Cystatin M/E was first reported as a potent inhibitor of papain (Ki = 0.39 nM) [28] and was also proposed as an inhibitor of peptidase 1 (C1 family) from the house dust mite (Dermatophagoides pteronyssinus) [37]. Cystatin M/E is primarily a tight-binding inhibitor of closely related human CatV and CatL endopeptidases (~80% protein sequence identity) (Table 1) and to a lesser extent CatB (Ki = 32 nM, [28]), but it does not inhibit CatC aminopeptidase [35,38,39]. 

### 3.2. Legumain (Asparaginyl Endopeptidase, AEP)

**Figure 1:**
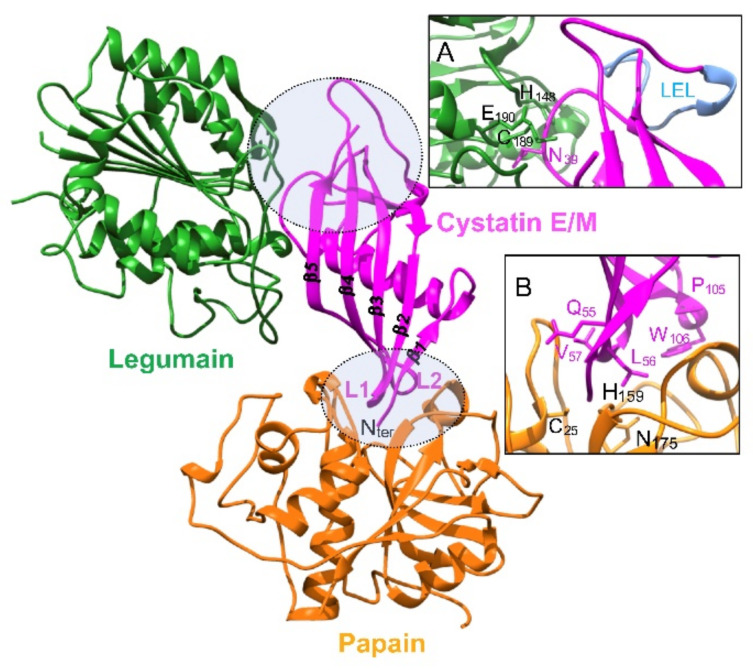
Structural schema representing cystatin M/E complexed with papain and human legumain. A model of cystatin M/E complexed with papain was performed using the protein structure homology-modelling Swiss-Model server (https://swissmodel.expasy.org/interactive) (accessed date: 31 January 2021). The structure of tarocystatin-papain (PDB: 3IMA.1) was used as a template (79.19% sequence identity of tarocystatin with cystatin M/E). The modeled cystatin M/E structure was then superimposed with the crystal structure of legumain in complex with cystatin M/E (PDB: 4N6N) [36]. Inhibitory sites of cystatin M/E are indicated by a circle. Inset A: zoom-in view of the legumain-interacting inhibitory loops: reactive center loop (RCL, including residue N39) and exosite loop (LEL). Inset B: zoom-in view of the inhibitory segments L1 (Q55LVAG59) and L2 (P105W106) of cystatin M/E with the active site of papain (catalytic triad: C25, H159, and D175). Key residues are shown in stick representation. Cystatin C numbering is used for cystatin M/E.

Cystatin M/E demonstrates a compelling affinity (Ki = 0.0016 nM) for pig legumain [10]. It appears to be the most effective physiological legumain inhibitor, binding more tightly as compared with cystatin C (Table 1). Among type 2 cystatins, cystatin C is also an effective inhibitor of legumain (Ki = 0.2 nM), while cystatin F displays a weaker potency (Ki = 10 nM). Conversely, cystatin D as well as stefins A and B (type 1 cystatins) and LK (type 3 cystatin) did not inhibit legumain. Legumain is a caspase-related protease (clan CD, family C13). As elegantly mentioned by Dall and Brandstetter [11], “legumain resembles a caspase in cathepsin’s clothing”. The legumain crystal structure confirmed its caspase-like fold [40]. Legumain harbors a strict substrate specificity, cleaving exclusively after Asn residue (P1 position), despite Asp being also tolerated at low pH conditions [41]. Crucially, legumain holds a unique dual protease-ligase activity, associated with a pH-dependent reversible on-off switching [36], which could lead to biologically relevant functional amendments with respect to its regulation by cystatins, in particular cystatin M/E. Site-directed mutagenesis of cystatin M/E revealed that Asn39 is crucial for the inhibition of legumain but not for CatL and CatV, albeit Trp106 was identified to be a key residue to inactivate CatV but not legumain (cystatin C numbering). Data support that legumain and papain-like cysteine proteases are inhibited by two distinct non-overlapping sites [35] and correlate with a seminal study that demonstrated the formation of a stable ternary complex between legumain, cystatin C and papain [10]. The crystal structure of cystatin M/E bound to its protease target was further deciphered and confirmed kinetics data [36]. While the papain-interacting region is composed of the N-terminal substrate-like region and L1 and L2 loops, cystatin M/E interacts with legumain according to a totally different mechanism [6] (Figure 1). 

The binding of cystatin M/E to legumain is mediated by a reactive center loop (RCL) according to a canonical, substrate-like binding mode with Asn39 on RCL serving as P1 residue and stabilized via exosite interactions by the so-called legumain exosite loop (LEL), which makes contact in primed substrate binding sites [36]. Cystatin M/E also inhibits auto-activation of secreted prolegumain, supporting a regulatory role in controlling both intra- and extracellular legumain activity [42]. Both glycosylated and unglycosylated cystatin M/E forms exhibit a similar legumain inhibitory potency, endorsing that the inhibition of legumain does not rely on its glycosylation status [32]. 

While it was shown that MDA-MB-435S, A375 and C8161 cells internalized substantial amounts of cystatin C, a low internalization of cystatin M/E was observed. Nevertheless, it may significantly affect intracellular legumain activity and cell migration [43]. On the other hand, secreted cystatin M/E does not reveal consistent effects on Fas-induced apoptosis [17]. Recently, it was shown that a domain-swapped dimer of cystatin M/E retained its capacity to inhibit legumain while binding sites toward papain-like proteases were buried. Alternatively, cystatin M/E amyloid fibrils retain the functional ability to inhibit both legumain and papain under in vitro conditions [44].

## 4. Biological Roles 

### 4.1. Cystatin M/E in Skin Homeostasis and Skin Disorders

Under biological conditions, the expression pattern of human cystatin M/E is distinctive among type 2 cystatins, as elevated protein levels are chiefly restricted to epidermal keratinocytes, hair follicles, sebaceous glands and sweat glands [38,45]. The role of cystatin M/E in skin has been outstandingly summarized elsewhere (see for review: [46]). Briefly, cystatin M/E colocalizes specifically with CatV in the stratum granulosum and in the root sheets of the hair follicle. Moreover, cystatin M/E is found in the extracellular space in the stratum corneum associated with corneodesmosomes, where it is thoroughly associated with CatV [47]. Cystatin M/E may directly control the activity of cysteine proteases, and both CatV-cystatin M/E and CatL-cystatin M/E balances have a critical regulatory role for epidermal differentiation, hair follicles, desquamation process of the stratum corneum and skin barrier formation, as validated by genetic ablation of cystatin M/E [46,48]. Accordingly, human *CST6* deficiency prevents the development of a multilayered epidermis in a 3D reconstructed skin model and may be incompatible with normal human foetal development [49]. Cystatin M/E also regulates the crosslinking of structural proteins by governing the CatL-dependent processing of transglutaminase 3 during the cornification process of the epidermis and the hair follicle [38,46]. A recent report showed that interleukin-17A downregulated the gene expression of *CST6* and profilaggrin, a major protein of keratohyalin granules in the granular layer [50]. Moreover, both CatL and CatV together with cystatin M/E may participate in cell shedding, which is associated with proteolytic impairment of skin adhesive structures, and notably of desmogleins [51].

Cystatin M/E deficiency in mice causes disturbed epidermal cornification, impaired barrier function and neonatal lethality because of excessive trans-epidermal water loss (for review: [46]). Harlequin ichthyosis is a relentless congenital skin disorder that usually leads to a stillborn fetus or neonatal death. Its clinical features at birth include a thickened fissured epidermis and are characterized by excessive epidermal and follicular hyperkeratosis. Concordantly, a null mutation in the cystatin M/E gene of harlequin ichthyosis (ichq) mice, which recapitulates most of the features of the human disease, was identified to be responsible for juvenile lethality and defects in epidermal cornification and desquamation [31]. Harlequin ichthyosis is associated with an unlimited activity of its target protease legumain in hair follicles and epidermis, leading to a dysregulation of legumain-cystatin M/E balance and an enhanced crosslinking of loricrin monomers by transglutaminase 3 [52]. Interestingly, the rescue of the skin ichq phenotype by transgenic, epidermis-specific, re-expressed cystatin M/E murine model does not completely restore the wild-type phenotype. Indeed, mice survive the neonatal phase, but display severe eye pathology and alopecia [53]. During chronic hand eczema, the desquamation-related kallikrein-related peptidases (KLK5 and KLK7) are downregulated, like filaggrin and cystatin M/E [54]. Inflammatory skin disorders (atopic dermatitis and psoriasis) also cause an extended expression of cystatin M/E, which is constitutively found in the stratum granulosum of normal skin and in the spinous cell layers (stratum spinosum) where it colocalizes with transglutaminase for which it serves as a substrate [55]. Yet, a decrease in both transcription and translation of cystatin M/E and CatV was reported in lesional atopic dermatitis and psoriasis epidermis. Conversely, mRNA levels of CatL and transglutaminase 3 were increased [45]. A homozygous variant, resulting in a stop codon in exon 2 of *CST6*, was associated with hypotrichosis, eczema and impaired sweating. Functionally, the corresponding recombinant mutant cystatin M/E protein lacks its ability to inhibit CatV, CatL and legumain [56]. Interestingly, analysis of the murine skin proteome showed that the deletion of CatL induced increased levels of acidic CatD, stefin B and cystatin M/E, and accumulation of the extracellular glycoprotein periostin [57].

Alternatively, Lyme disease results from infection with the spirochete Borrelia burgdorferi. The first and common clinical manifestation, called erythema migrans, corresponds to a circular, inflamed skin lesion. Proteomic analysis was conducted on an independent cohort of patients with erythema migrans, leading to the identification of some candidate molecules, including cystatin M/E, as putative acute-phase blood markers for the diagnosis of early Lyme disease [58].

### 4.2. Cystatin M/E and Reproductive System

In an early report, it was proposed that cystatin M/E in the amniotic fluid may participate in fetal development [28]. During the estrous cycle, *CST6* was identified as a pig pregnancy-related gene and dynamic changes in its mRNA level were observed in bovine endometrium [59,60]. On the other hand, interferon tau and prostaglandins, which are key players of the regulation of the peri-implantation period of pregnancy in sheep, increase expression of endometrial cystatin M/E, which in turn could play a role in tissue remodeling, in placental and fetal development and drive conceptus elongation in beef heifers [60,61,62]. Cystatin M/E, which is expressed at the maternal-fetal interface together with legumain, may participate in the establishment and maintenance of pregnancy in pigs [63]. Additionally, analysis of the ovarian transcriptome suggested that the differentially expressed *CST6* gene could be a candidate gene for prolificacy traits in sheep [64]. 

### 4.3. Cystatin M/E in Neurodegenerative Progressive Diseases and Atherosclerosis

Type 1 cystatins and cystatin C were proposed to play a role in Alzheimer’s disease according to their ability to form amyloid fibrils [65,66,67]. Interestingly, the conformational destabilization of cystatin M/E leads to the formation of a domain-swapped dimer, still acting as an effective legumain inhibitor while cathepsin binding sites are buried. Then, cystatin M/E dimers may further convert to amyloid fibrils [44]. Aside from being an oncogene, DJ-1 was also identified as a syndrome-associated protein of a familial form of Parkinson’s disease, working as a coactivator to diverse transcription factors. An increased level of cystatin M/E was detected in the cerebrum, spleen and heart from DJ-1-deficient mice, leading to a decreased activity of legumain, which participates in the DJ-1-regulated activation and cleavage of annexin A2 [68]. Elsewhere, an unsTable 15- to 18-mer minisatellite repeat expansion in the promoter region of *CST6* was identified as a disease-causing mutation of progressive myoclonus epilepsy of Unverricht-Lundborg type, a rare autosomal recessive disorder [69]. Yet, a rat homolog of cystatin M/E, which was expressed by embryonal hippocampal cells, was proposed to contribute to neuronal cell differentiation [70]. Together with cystatins C and M/E, legumain expression is increased in both plasma and plaques of patients with carotid stenosis and was proposed as an early biomarker of atherosclerosis [71].

## 5. Cystatin M/E: Tumor Suppressor and Biomarker in Breast Cancer

In a seminal in vitro study, cystatin M was identified as a downregulated transcript in a metastatic breast cancer cell line when compared to a matched primary tumor cell line. Consequently, loss of cystatin M/E expression was proposed to be associated with the progression of human breast cancer [27]. Transfection of cystatin M/E in highly tumorigenic and metastatic human breast cancer cells (MDA-MB-435S cell line) impaired proliferation, migration, matrix invasion and tumor-endothelial cell adhesion, providing the first evidence that cystatin M/E plays a protective role in safeguarding against breast cancer [72]. Consistently, SCID (severe combined immunodeficiency) mice that have been orthotopically implanted with breast cancer cells expressing cystatin M/E revealed a delayed primary tumor growth and lower metastatic burden in the lung and liver [73,74]. Conversely, deficiency of cystatin M/E expression was reported in invasive ductal carcinoma cells from stage IV patients compared to normal human breast epithelial cells, sustaining that cystatin M/E could be a candidate tumor suppressor gene [73]. An analysis of differential gene expression of triple-negative breast cancer (TNBC)-derived MDA-MB-231 cells demonstrated that *CST6* was also strongly repressed, in addition to type 2 cystatin genes (*CST1, CST2* and *CST4*), which are all encoded by a gene cluster on 20p11 [75].

Bone metastasis is a common cause of morbidity and mortality in breast cancer patients. Jin and colleagues demonstrated that *CST6* was downregulated in bone metastatic (MDA-MB-231) derivative cell lines. Ectopic expression of *CST6* suppressed proliferation, colony formation, migration and invasion in vitro. This overexpression rescued mice from osteolytic metastasis and death, while *CST6* knock-down markedly enhanced cancer cell bone metastasis and shortened animal survival, sustaining that cystatin M/E is a bona fide suppressor of breast cancer osteolytic metastasis [76].

The loss of cystatin M/E expression has been strongly correlated with *CST6* promoter hypermethylation in a panel of breast cancer cell lines (MDA-MB-231, ZR75-1, MCF-7, T47D, BT-549, Hs578T, MDA-MB-436 and MDA-MB-453) and in primary breast carcinomas, indicating that this aberrant characteristic is common in breast malignancies [77,78,79]. Hypermethylation, which arises before the development of invasive breast cancer, occurs within the vicinity of a large and dense CpG-rich promoter island in the 5′ region of *CST6* [79,80]. An aberrant methylation profile of *CST6* was also found in axillary lymph node metastasis in comparison to primary tumor tissue and adjacent normal tissue from the same breast cancer patients [81,82]. Treatment with a DNA demethylating agent (5-aza-2′-deoxycytidine, 5-aza-dC) reactivated *CST6* transcription and expression, substantiating that cystatin M/E is an epigenetically-inactivated tumor suppressor gene [77,78,79]. 

Basal-like breast tumors (~25% of all breast cancers) contribute disproportionately to breast cancer deaths, as they have the propensity to display aggressive tumor characteristics (increased size, rapid tumor growth, increased rate of metastasis, higher incidence of relapse and lower overall patient survival). A cluster of tumors, identified as basal-like breast cancers, expresses a hypermethylator signature with a low expression level of six specific genes including *CST6* and is associated with an aberrant overexpression of DNA (cytosine-5)-methyltransferase 3β (DNMT3b) [83]. Secondary to DNMT3b overexpression, the molecular mechanism governing DNMT3b gene-mediated aberrant DNA hypermethylation involves the loss of post-transcriptional regulation of DNMT3b by regulatory microRNAs [84].

The prognostic significance of the methylation status of *CST6* as a cancer biomarker in breast tumors was statistically evaluated. Multivariate analysis showed that *CST6* promoter methylation status was an independent prognostic factor for disease-free-interval (DFI) and overall survival (OS) [85]. Data support that *CTS6* hypermethylation may serve as a biomarker for clinical molecular diagnosis and is associated with unfavorable prognosis in operable breast cancer [85,86]. The *CST6* promoter methylation status of circulating tumor cells (CTCs) isolated from peripheral blood of breast cancer patients was also analyzed. *CST6* hypermethylation for both operable and metastatic breast cancer significantly differed from that of the control population [87]. Moreover, metastatic breast cancer patients with CTCs unmethylated for *CST6* have a significantly longer progression-free survival compared to patients with corresponding enriched methylated CTCs [88]. A panel of 13 genes, including *CST6*, may enable breast cancer prediction and patient stratification, based on well-defined DNA methylation profiles, and might be a valuable biomarker for early detection of breast cancer [89]. In a pilot study, it was observed that *CST6* promoter is hypermethylated in cell-free DNA circulating in the plasma of breast cancer patients, but not in healthy individuals. Data support that *CST6* promoter methylation in cell-free DNA could be a putative novel plasma tumor marker for breast cancer [90]. Alternatively, a clear association between the epithelial cell adhesion molecule (EpCAM)-positive CTC-fraction and circulating tumor DNA (ctDNA) for promotor methylation of the transcriptional regulator SOX17, but not for *CST6*, was found for both patients with early and metastatic breast cancer [91]. At the molecular level, this hypermethylation is associated with aberrant serine/threonine kinase AKT1 activation in epithelial cells, as well as the disabled inositol polyphosphate-4-phosphatase, type II (INNP4B) regulator that results from suppression by cancer-associated fibroblasts. Accordingly, microenvironmental stimuli may trigger this epigenetic cascade and lead to the downregulation of cystatin M/E in breast tumors [92]. Moreover, hypermethylation of the *CST6* gene is influenced by the alteration of estrogen receptor (ER) and receptor tyrosine-protein kinase erbB-4 (HER4), and cystatin M/E was identified as a downstream target of ER and HER4 [93].

Cystatin M/E may modulate malignant properties of human breast carcinoma cells (MDA-MB-435S cells) partly through the downregulation of two major secreted signaling molecules, autotaxin/lyso-phospholipase D and interleukin-8, which combine mitogenic, motogenic and angiogenic properties [94]. *CST6* was identified as a repressed TBX2 (a T-box family oncogenic transcription factor) target gene through a mechanism involving Early Growth Response 1 (EGR1). An increase in TBX2 expression is known to drive breast cancer proliferation and metastasis. In contrast, the exogenous expression of cystatin M/E in TBX2-expressing breast cancer cells resulted in upregulated apoptosis. Remarkably, induction of apoptosis depends on the cystatin M/E legumain-inhibitory domain, but not on the cysteine cathepsin-inhibitory domain, emphasizing that legumain is a crucial oncogenic driver. Secretion or glycosylation of cystatin M/E is not a prerequisite to elicit cell killing effects. Thus, cystatin M/E represents an important barrier to breast tumorigenesis which could be by-passed by TBX2 transcriptional repression. Data also sustain that the “TBX2-cystatin M/E-legumain” pathway may represent a novel promising axis for the development of innovative therapies to target TBX2-driven breast cancers [95]. It could be noticed that *CST6* was also repressed through a mechanism involving both TBX2 and EGR1 in rhabdomyosarcoma, a pediatric malignant cancer which is thought to arise from myogenic precursors in the skeletal muscle lineage [96].

## 6. Cystatin M/E: Tumor Suppressor and Biomarker in Other Cancers

### 6.1. Lung Cancer

Type I cystatins were proposed as independent prognostic markers for patients with non-small cell lung cancer (NSCLC), since high levels of both stefins A and B, but not cystatin M/E, are associated with a better survival probability. Compared to cystatin C (median concentration: 3.7 ng.mg^−1^ of protein), the estimated median level of cystatin M/E in NSCLC primary tumors is 14.6 pg.mg^−1^ of protein [97]. On the other hand, *CST6* is frequently transcriptionally downregulated in NSCLC tumors, while its ectopic expression suppresses NSCLC growth in culture [98]. Transcriptomics of lung adenocarcinoma cell lines derived from both smokers (S) vs. never smokers (NS) demonstrated that the methylation of *CST6* gene differed significantly based on smoking practice [99]. Like cystatin C, immunoreactive cystatin M/E levels (median concentration into the pleural space: 0.14 nM) are significantly higher in effusions of primary pleural tumors (mesotheliomas) compared to secondary pleural tumors (3.4 μg/L vs. 2.5 μg/L) [22].

### 6.2. Cervical Cancer

The loss of expression of cystatin M/E that is associated with *CST6* inactivation by somatic mutations and promoter hypermethylation was also detected in primary cervical tumors. Interestingly, expression of cystatin M/E was recovered after 5-aza-dC and/or trichostatin A treatment, while its ectopic expression in cell lines resulted in growth suppression, supporting that cystatin M/E is a cervical cancer suppressor [100]. An inverse correlation between the expression of cystatin M/E and CatL was detected in cervical carcinoma and primary tumors. On the other hand, a direct relationship between the deficiency of cystatin M/E and the regulation of the nuclear factor-kappa B (NF-κB) signaling pathway and its nuclear expression was reported, suggesting that cystatin M/E participated in the reduction of tumor cell growth through cytoplasmic retention of NF-κB [101]. 

### 6.3. Brain and Neck Cancer

Malignant gliomas are the most common primary brain tumors in adults and the second most common tumor in children, and are characterized by a high morbidity and mortality. It was found that *CST6* promoter was hypomethylated in normal brain samples, albeit most primary brain tumors demonstrated deficient cystatin M/E expression, in agreement with *CST6* promoter hypermethylation. Moreover, *CST6* expression by glioma tumor initiating cells (TIC) was fully hampered by promoter methylation, while the ectopic expression of cystatin M/E reduced TIC motility and invasion. Qiu et al. [102] proposed that analysis of *CST6* epigenetic silencing may therefore represent a novel prognostic marker of gliomas. Interestingly, together with *CST6*, the apoptosis-inducer Bcl-2-interacting killer (BIK) is also a target of epigenetic silencing in malignant gliomas. BIK, which is a member of the BH3-only family of pro-apoptotic proteins, is known to suppress glioma cell growth in culture [103]. The treatment of SCC-25 neck squamous carcinoma cells with a pleiotropic calcemic vitamin D3 analog (EB1089) lead to a 20-fold increase in cystatin M/E expression compared to a control [104].

### 6.4. Cutaneous Squamous Cell Carcinoma and Melanoma

Conversely to benign hyperplasia as observed during psoriasis vulgaris, a unique cancer-specific gene expression signature was identified for human cutaneous squamous cell carcinoma (cSCC) associated with a gene downregulation of granzyme B, and a differential gene upregulation (fold change > 3) of matrix metalloproteinase MMP-1, kallikrein 7, CatV and cystatin M/E [105]. Contrariwise, proteome profiling of primary and metastatic cSCC lesions by mass spectrometry-based proteomics demonstrated a decrease in cystatin M/E in metastatic cSCC lesions relative to the primary phenotypes (fold change = −3.9), and the authors suggested that cSCC metastasis may be partly attributed to the decreased level of cystatin M/E in metastatic lesion phenotype [106]. Accordingly, further investigation should be conducted to clarify the regulation of *CST6* and cystatin M/E protein expression, as well as its pathophysiological (deleterious vs. protective) relevance in cSCC metastasis. On the other hand, *CST6* is involved in the suppression of proliferation, migration and metastasis of melanoma [107]. Interestingly the glycosylated ~17 kDa form of cystatin M/E, but not the non-glycosylated ~14 kDa form, is predominantly detected in melanoma cell lines secreting cystatin M/E, while cystatin C is lacking or expressed at a very low level. Invasion is suppressed in cystatin M/E overexpressing melanoma cell lines and intracellular legumain activity is drastically impaired. Conversely, CatB activity is not affected, which suggests that cystatin M/E specifically regulates legumain activity and therefore the invasive potential of human melanoma cells [108]. Additionally, in the tumor acidic microenvironment, secreted cystatin M/E could prevent auto-activation of secreted prolegumain. Alternatively, in its absence, extracellular prolegumain could auto-activate and further cleave target substrates (e.g., fibronectin), thus promoting tumor invasion and metastasis [42]. 

### 6.5. Prostate and Gastric Cancer

Expression of cystatin M/E is extensively decreased in metastatic prostate cell lines and prostate tumor tissues compared to normal human prostate epithelium [109]. Treatment of metastatic prostate cell lines with a histone deacetylase inhibitor transcriptionally upregulates *CST6* and increases cystatin M/E protein expression. Its overexpression significantly reduces prostate cell proliferation and inhibits tumor growth and the incidence of lung metastasis. The authors proposed that *CST6* downregulation was associated with promoter histone modifications and may have participated in prostate cancer progression during the invasive and metastatic stages. Analysis of the methylation status of *CST6* in gastric carcinomas and their paired adjacent non-tumor tissues revealed a loss of expression of cystatin M/E in 70% of gastric carcinomas due to promoter hypermethylation. Moreover, patients with hypermethylated *CST6* had a significantly shorter survival time than those with hypomethylated promoter [110]. In a close way, a CpG island methylator phenotype-related prognostic gene signature, including *CST6*, may allow the classification of patients with gastric cancer into high-and low-risk groups with a significant OS difference [111].

### 6.6. Renal Carcinoma

Epigenetic study by high-density gene expression microarrays of renal cell carcinoma (RCC) cell lines identified eight genes, including *CST6*, exhibiting tumor-specific promoter region hypermethylation associated with transcriptional silencing [112]. Hypermethylation of *CST6* is associated with both a shortened progression-free and OS of patients with metastasized RCC undergoing anti-vascular endothelial growth factor (VEGF)-based therapy [113]. Following transfection and subsequent expression of cystatin M/E, the growth of RCC cell lines was compromised, supporting the status of cystatin M/E as a candidate kidney tumor suppressor [112] (summarized in Table 2).

## 7. Tumor-Promoting Role of Cystatin M/E

### 7.1. Triple-Negative Breast Cancer

Besides its tumor-suppressive function, cystatin M/E transcripts were detected in both primary and metastatic breast cancer cells. Increased expression of cystatin C and cystatin M/E, which correlated significantly with a larger tumor size, was observed in breast cancer cells isolated by laser capture microdissection [114]. Analysis of patients suffering from TNBC (10–20% of human ductal adenocarcinomas), which is the most aggressive breast cancer type, with the poorest prognosis and highest mortality rates, demonstrated that cystatin M/E was unexpectedly elevated in TNBC tissues vs. adjacent normal breast tissues. Moreover, increased levels of cystatin M/E correlated with a high risk of lymph-node metastasis and a low disease-free survival rate, supporting that cystatin M/E could be used as an independent predictor of disease-free survival in TNBC. Conversely to the archetypal role of cystatin M/E as a tumor suppressor in breast cancer, the authors suggested that cystatin M/E may be involved in the pathophysiology of TNBC, possibly acting as a tumor-promoting molecule [115]. Nevertheless, these unanticipated results need to be further confirmed.

### 7.2. Oral Cancer

Analysis of cell lines derived from primary and metastatic lesions of oropharyngeal squamous cell carcinomas indicated that cystatin M/E was expressed 40-fold higher in the metastatic vs. the primary tumor cell line. Unexpectedly, upregulation of cystatin M/E may favor metastasis by blocking intrinsic CatB activity and rescuing tumor cells from TNF-α-induced apoptosis [117]. The silencing of *CST6* expression in tumor oral cancer cell line increases both invasion and motility, but also renders metastatic MDA-686Ln cells hyperproliferative [118].

### 7.3. Pancreatic Cancer

No or limited expression of cystatin M/E was observed in normal pancreas, while immunohistochemical analysis confirmed cystatin M/E overexpression in pancreatic ductal adenocarcinoma (PDAC). Its constitutive expression in *CST6*-null cells promotes their growth in vitro and in vivo, and the addition of exogenous active cystatin M/E, but not recombinant cystatin M/E lacking its proteinase-inhibitor domain, promotes cell proliferation. Conversely, knock-down of cystatin M/E attenuates PDAC cell growth. The authors endorse that cystatin M/E may be involved in the proliferation and survival of pancreatic cancer, probably through the inhibition of intracellular pro-apoptotic CatB [119].

### 7.4. Papillary Thyroid Carcinoma

*CST6* is induced subsequently to serine/threonine-protein kinase B-Raf (BRAF) activation and therefore may be downstream in the BRAF/MEK (mitogen-activated protein kinase kinase)/extracellular signal-regulated kinase signaling pathway in papillary thyroid carcinoma (PTC) [120]. Moreover, *CST6*, together with C-X-C motif chemokine ligand 14 (CXCL14) and secreted phosphoprotein 1 (SPP1, osteopontin), is associated with PTC lymph node metastasis. 

### 7.5. Hepatocellular Carcinoma 

Survival analysis indicated that *CST6* correlated with the OS and recurrence-free survival (RFS) of patients with hepatitis B virus-related hepatocellular carcinoma (HBV-related HCC) [116]. *CST6* may function as a prognostic biomarker for HCC, with a high *CST6* expression being associated with poor survival of patients, in correlation with a previous report claiming that cystatin M/E could be a pancreatic tumor promotor [119]. Taken together, data suggest that cystatin M/E may participate in diverse molecular mechanisms and exert distinct effects in different types of cancer [116].

## 8. Conclusions

Cystatin M/E (type 2 cystatin) is a specific and dual tight-binding inhibitor of both cathepsins L, V and B (family C1) and legumain (or asparaginyl endopeptidase, family C13), via two distinct mechanisms of inhibition, which involve non-overlapping binding sites. Cystatin M/E plays a critical role in epidermal homeostasis, and its deregulation is associated with various skin diseases such as ichthyosis or psoriasis. Cystatin M/E is also probably involved in fetal and placental development and could participate in the pathogenesis of neurodegenerative progressive diseases, according to its ability to form amyloid fibrils. *CST6* was initially identified as a tumor suppressor and an epigenetically downregulated gene in breast cancer. The methylation status of *CST6* holds a prognostic significance and may also serve as a biomarker for clinical diagnosis. Afterward, this tumor-suppressing role was corroborated with other cancers (cutaneous carcinoma, melanoma, lung, cervical, brain, neck, prostate, gastric and renal cancers) and the use of *CST6* as a valuable biomarker was confirmed. Conversely, increased levels of cystatin M/E in TNBC tissues correlate with a higher risk of metastasis and a lower disease-free survival rate. Thus, beside its conventional tumor-suppressing role, cystatin M/E may act as a tumor-promoting effector. This outward antagonistic function was further testified for thyroid, oral and pancreatic cancer and hepatocellular carcinoma. Accordingly, molecular and cellular analysis of the regulatory mechanisms of the expression and activity of cystatin M/E associated with the maintenance of the proteolytic balance, as well its imbalance under pathophysiological conditions, has to be urgently deepened. Moreover, the putative involvement of cystatin M/E, during tumorigenesis, in signaling pathways independent of its protease inhibitor function, still remains to be deciphered.

## Figures and Tables

**Table 1:** Inhibitory constant (Ki) of human cystatins for human target proteases.

	Legumain	Cathepsin V	Cathepsin L
Cystatin M/E		Ki (nM) *	
Wild-type	0.25 (0.0016)	0.47	1.78
N39A mutant	>100	0.34	1.77
W106A mutant	0.72	>100	11.7
Cystatin C	7.28 (0.2)	0.02	0.08
Cystatin D	>100 (>1000)	29.6	2.81
Cystatin F	>100 (10)	>100	0.49
Cystatin S	>100	>100	>100
Stefin A	>100 (>1000)	0.11	0.05
Stefin B	>100 (>1000)	0.13	0.05

For the sake of homogeneity with the body text, cystatin C numbering is used. * From [35]. In bracket: pig legumain [10].

**Table 2:** Roles of cystatin M/E (*CST6*) in cancer—a short summary.

Tumor Type	Tumor-Suppressive	Tumor-Promoting	References
Breast cancer	Ectopic expression of *CST6* reduces cell growth, proliferation, migration, invasion in vitro and in vivo. Patients with methylated *CST6* have a worse disease-free and overall survival rate	Increased expression in breast cancer cells. Overexpression of cystatin M/E in TNBC tissues is associated with a low disease-free survival rate	[72,73,74,85,86,114,115]
Cervical cancer	Loss of cystatin M/E in primary tumors. *CST6* re-expression inhibits tumor cell growth		[100]
Cutaneous squamous cell carcinoma (cSCC)	cSCC metastasis is partly attributed to a decrease in cystatin M/E expression	Overexpression of *CST6* in cSCC tissues vs adjacent normal skin	[105,106]
Gastric cancer	Shorter survival time for patients with methylated *CST6* promoter		[110,111]
Glioma	Reduction of cell motility and invasion after *CST6* overexpression		[102]
Hepatocellular carcinoma		Increased cystatin M/E expression is correlated with poor survival of HCC patients	[116]
Lung cancer	*CST6* ectopic expression suppresses NSCLC cell lines growth		[98]
Melanoma	*CST6* involved in the suppression of proliferation, migration and metastasis of melanoma cells		[42,107,108]
Oral cancer		*CST6* is overexpressed in oropharyngeal metastatic cells. Its silencing increases cell proliferation and invasion	[117,118]
Pancreatic cancer		Cystatin M/E expression is associated with PDAC cells growth and proliferation	[119]
Papillary thyroid carcinoma (PTC)		Increase of *CST6* in PTC is associated with lymph node metastasis	[120]
Prostate cancer	Overexpression of cystatin M/E inhibits cell growth and lung metastasis incidence		[109]
Renal cell carcinoma (RCC)	Promoter hypermethylation of *CST6* is associated with a shortened progression-free and overall survival of patients with metastasized RCC		[112,113]
